# Markers of insulin resistance associated with non-alcoholic fatty liver disease in non-diabetic population

**DOI:** 10.1038/s41598-023-47269-4

**Published:** 2023-11-22

**Authors:** Pei Zeng, Xiangsheng Cai, Xiaozhou Yu, Linjing Gong

**Affiliations:** 1 Guangzhou Cadre Health Management Center, Guangzhou, 510000 China; 2 School of Public Health, Sun Yat-Sen University, Guangzhou, 510000 China

**Keywords:** Endocrinology, Medical research

## Abstract

Insulin resistance (IR) plays an important role in the development of non-alcoholic fatty liver disease (NAFLD). IR markers are divided into two types: (1) insulin-based IR marker, homeostatic model assessment of IR (HOMA-IR); and (2) non-insulin-based IR markers, such as triglyceride-glucose (TyG) index, TyG index with body mass index (TyG-BMI), triglyceride/high-density lipoprotein cholesterol ratio (TG/HDL-c), and metabolic score for IR (METS-IR). The non-insulin-based IR markers are often associated with lipids. The aim of this study was to analyse the association between IR markers and NAFLD in non-diabetic population. Baseline data of NAFLD and non-NAFLD groups were compared. Logistic regression was used to evaluate the relationship between five IR markers and NAFLD risk. The odds ratios (ORs) and 95% confidence intervals (CIs) of IR markers were calculated. Receiver operating characteristic (ROC) curves and area under the curve (AUC) were used to evaluate the ability of different IR markers to detect NAFLD. Subgroup analyses were performed in obese and non-obese subgroups. This study found a positive correlation between NAFLD risk and elevation in five IR markers (HOMA-IR, TyG, TyG-BMI, TG/HDL-c, and METS-IR). In non-obese subjects, the AUC of TyG-BMI was larger than that of the other four IR markers to detect NAFLD. The AUC of HOMA-IR was larger than that of the other four IR markers to detect NAFLD in obese subjects. In non-diabetic population, the five IR markers are associated with the risk of NAFLD, including non-obese and obese NAFLD. TyG-BMI and HOMA-IR can be used to detect non-obese and obese NAFLD, respectively, with better detection ability compared with the other IR markers.

## Introduction

The global prevalence of non-alcoholic fatty liver disease (NAFLD) is around 25%^1^, with an increasing trend observed in China^2^. It is often associated with metabolic comorbidities^3^. Age is also considered a risk factor^4^. Although NAFLD is typically accompanied by obesity, it can also occur in individuals who are not obese, which is known as non-obese NAFLD. Subjects with non-obese NAFLD appear to have clinical outcomes comparable to or worse than subjects with obese NAFLD^5,6^. Non-obese NAFLD may be more prevalent in Asia than in other regions^7^. Because the indication for screening for non-obese NAFLD is unclear, patients may be easily missed when they do not fit the classic phenotype of obesity. Liver biopsy is the gold standard for diagnosis and staging of NAFLD, but it is limited by its invasiveness. Therefore, there is an urgent need for techniques to detect NAFLD, especially non-obese NAFLD, in order to early identify specific populations at risk for the disease.

Currently, diagnostic methods, such as ultrasound (US), computed tomography (CT), and magnetic resonance imaging (MRI) are routinely used to screen NAFLD. However, the high cost of CT and MRI is a limiting factor. US is relatively inexpensive but poorly sensitive for mild steatosis^8^. Moreover, US is often reported independently by a single physician, and diagnostic differences may exist between physicians.

Insulin resistance (IR) plays an important role in the development of NAFLD^9,10^. Baseline insulin resistance is associated with the progression of liver fibrosis in non-diabetic patients with NAFLD^11^. Therefore, IR markers may be the first step in early detection of the disease in non-diabetic populations.

The hyper-insulinemic euglycemic clamp is the gold standard for assessing insulin sensitivity^12^, but the cost and complexity limit its clinical application. Several studies have reported methods to predict IR, Commonly used IR markers including homeostatic model assessment of IR (HOMA-IR)^13,14^, triglyceride-glucose (TyG) index, TyG index with body mass index (TyG-BMI), triglyceride/high-density lipoprotein cholesterol ratio (TG/HDL-c), and metabolic score for IR (METS-IR)^15^. HOMA-IR is based on insulin levels. TyG, TyG-BMI, TG/HDL-c, and METS-IR are independent of insulin levels and are based on lipid parameters and related indicators^15^. This study analysed the association between the five IR markers and NAFLD in non-diabetic population. It also compared the predictive ability of the five IR markers for obese and non-obese NAFLD for large-scale screening of the population.

## Methods

### Study population

A total of 2148 subjects who underwent physical examination were randomly selected for screening between 2021 and 2023. The participants had no previous history of diabetes or hepatitis. The participants’ sex ratio was similar to that of the subjects who underwent physical examination in our hospital.

Exclusion criteria were: (1) a history of diabetes or taking diabetes medication or fasting plasma glucose (FPG) ≥ 7.0 mmol/L; (2) a history of viral hepatitis, autoimmune hepatitis, hepatic malignancy, drug-induced liver disease, or hepatolenticular degeneration; (3) a history of excessive drinking; (4) severe liver or kidney insufficiency; (5) patients undergoing treatment with lipid-regulating drugs; and (6) incomplete clinical data.

Each patient’s name, gender, age, current and past medical history, and recent medication information were collected. Venous blood was collected in the morning after the subject had fasted for at least 10 h. Levels of total cholesterol (TC), high-density lipoprotein cholesterol (HDL-c), low-density lipoprotein cholesterol (LDL-c), triglycerides (TG), and FPG, aspartate aminotransferase (AST), alanine aminotransferase (ALT), gamma glutamyl transpeptidase (GGT), uric acid (UA), and fasting insulin (FINS) were determined with Canon TBA-FX8 Automated Biochemical Analyzer (Japan).

BMI was calculated as weight (kg) divided by height squared (m^2^). Based on BMI, the subjects were categorised into non-obese (BMI < 25 kg/m^2^) and obese (BMI ≥ 25 kg/m^2^)^16^ subjects. HOMA-IR, TyG, TyG-BMI, TG/HDL-c, and METS-IR were calculated as follows^15,17,18^:

$${\text{HOMA-IR }} = {\text{ FPG }}\left( {{\text{mmol}}/{\text{L}}} \right) \, \times {\text{ FINS }}\left( {\upmu {\text{U}}/{\text{mL}}} \right)/{22}.{5},$$

$${\text{TyG }} = {\text{ ln}}[{\text{TG}}\left( {{\text{mg}}/{\text{dL}}} \right) \times {\text{FPG}}\left( {{\text{mg}}/{\text{dL}}} \right)/{2}],$$

$${\text{TyG-BMI }} = {\text{ TyG}} \times {\text{BMI}},$$

$${\text{TG}}/{\text{HDL-c }} = {\text{ TG }}\left( {{\text{mg}}/{\text{dL}}} \right)/{\text{HDL-c }}\left( {{\text{mg}}/{\text{dL}}} \right),$$

$${\text{METS-IR }} = {\text{ ln }}[({2} \times {\text{FPG }}\left( {{\text{mg}}/{\text{dL}}} \right)) \, + {\text{ TG }}\left( {{\text{mg}}/{\text{dL}}} \right)] \times {\text{BMI}})/\left( {{\text{ln }}\left[ {{\text{HDL-c }}\left( {{\text{mg}}/{\text{dL}}} \right)} \right]} \right).$$

All subjects were diagnosed with NAFLD by two experienced physicians using CT.

### Ethics approval and consent to participate

The study was performed in accordance with the Declaration of Helsinki. All experiments were approved and carried out following the guidelines of the Ethics Committee of Guangzhou Cadre Health Management Center (Approval Number: K2022-07). Informed consent was obtained from all participants.

### Statistical methods

Statistical analysis was performed using SPSS Statistics for Windows, version 26.0. Due to the skewed distribution of the data, continuous data were expressed as medians and interquartile ranges. Differences between dichotomous variables were analysed by chi-square test. Mann–Whitney U test was used to analyse the differences between the two groups of continuous variables. Multivariate logistic regression models with IR marker values as categorical variables were constructed based on quartiles of HOMA-IR, TyG, TyG-BMI, TG/HDL-c, and METS-IR in the overall population, non-obese, and obese subgroups, respectively. Regression model 1 was a rough model without adjustment. Model 2 was adjusted for age, sex, and history of hypertension to assess the relationship between the five IR markers and the risk of NAFLD. The odds ratio (OR) and 95% confidence interval (CI) for IR markers were calculated. Receiver operating characteristic (ROC) curves and area under the curve (AUC) were used to evaluate the predictive ability of different IR markers for NAFLD. A P value of < 0.05 defined statistical significance.

## Results

### Characteristics of participants

Compared with non-NAFLD group, the NAFLD group had significantly higher proportion of males and those with hypertension; Values of BMI, FPG, UA, TG, AST, ALT, GGT, HOMA-IR, TyG, TG/HDL-c, METS-IR, and TyG-BMI were significantly elevated. The results revealed 928 obese and 1220 non-obese subjects. The BMI of NAFLD group was significantly higher than that of non-NAFLD group in both obese or non-obese subgroups (Table 1).

**Table 1:** Comparison of baseline characteristics between groups with and without NAFLD.

	non-NAFLD group (n = 1794)	NAFLD group (n = 354)	P value
Age (years)	57 (53, 63)	58 (53, 61)	0.544
Male (%)	73.9	81.1	0.004
Hypertension (%)	18.9	35.0	< 0.001
BMI (kg/m^2^)	24.13 (22.35, 25.98)	26.25 (24.75, 27.98)	< 0.001
BMI of Non-obese (kg/m^2^)	22.82 (21.45, 23.96)	23.93 (23.20, 24.40)	< 0.001
BMI of obese (kg/m^2^)	26.50 (25.72, 27.69)	27.31 (25.98, 28.73)	< 0.001
FPG (mmol/L)	5.40 (5.10, 5.75)	5.69 (5.32, 6.12)	< 0.001
UA (μmol/L)	384.75 (321.40–446.18)	429.90 (373.88–499.88)	< 0.001
TG (mmol/L)	1.17 (0.86, 1.62)	1.70 (1.27, 2.52)	< 0.001
TC (mmol/L)	5.35 (4.68, 5.98)	5.23 (4.56, 6.01)	0.171
HDL-c (mmol/L)	1.39 (1.20, 1.63)	1.23 (1.09, 1.39)	< 0.001
LDL-c (mmol/L)	3.35 (2.74, 3.94)	3.29 (2.53, 3.89)	0.095
AST (U/L)	20.20 (17.50, 23.60)	22.10 (18.95, 27.73)	< 0.001
ALT (U/L)	20.00 (15.50, 26.90)	28.50 (22.08, 40.05)	< 0.001
GGT (U/L)	22.20 (16.50, 31.90)	30.85 (22.38, 42.60)	< 0.001
HOMA-IR	2.24 (1.65, 3.18)	3.92 (2.85, 5.52)	< 0.001
TyG	8.54 (8.20, 8.88)	8.98 (8.66, 9.38)	< 0.001
TG/HDL-c	1.90 (1.27, 2.87)	3.13 (2.19, 5.36)	< 0.001
METS-IR	34.85 (30.97, 38.71)	40.42 (37.29, 43.93)	< 0.001
TyG-BMI	207.24 (187.08, 226.14)	236.65 (221.16, 257.10)	< 0.001

The risk of NAFLD increased significantly with increasing TyG, TyG-BMI, HOMA-IR, TG/HDL-c, and METS-IR quartiles. Based on the ORs, we found that the positive effect of the highest quartile of TyG-BMI was most pronounced in the adjusted Model 2 (Table 2).

**Table 2:** Logistic regression analysis of 5 IR markers associated with NAFLD.

	Model 1		Model 2	
	P value	OR (95% CI)	P value	OR (95% CI)
HOMA-IR				
Q1		Ref		Ref
Q2	0.039	1.809 (1.030, 3.176)	0.049	1.761 (1.001, 3.096)
Q3	< 0.001	5.292 (3.207, 8.733)	< 0.001	5.033 (3.043, 8.323)
Q4	< 0.001	16.701 (10.343, 26.967)	< 0.001	15.425 (9.496, 25.058)
TG/HDL-c				
Q1		Ref		Ref
Q2	< 0.001	2.801 (1.685,4.657)	< 0.001	2.618 (1.568, 4.373)
Q3	< 0.001	5.837 (3.621, 9.408)	< 0.001	5.289 (3.262, 8.575)
Q4	< 0.001	11.298 (7.107, 17.959)	< 0.001	10.540 (6.565, 16.923)
TyG				
Q1		Ref		Ref
Q2	< 0.001	2.664 (1.598, 4.440)	< 0.001	2.565 (1.535,4.286)
Q3	< 0.001	5.237 (3.243, 8.458)	< 0.001	4.969 (3.068, 8.048)
Q4	< 0.001	12.308 (7.747, 19.556)	< 0.001	11.300 (7.079, 18.039)
METS-IR				
Q1		Ref		Ref
Q2	< 0.001	4.968 (2.393, 10.311)	< 0.001	5.567 (2.657, 11.666)
Q3	< 0.001	14.632 (7.324, 29.233)	< 0.001	17.111 (8.401, 34.853)
Q4	< 0.001	33.720 (17.051, 66.686)	< 0.001	39.055 (19.203, 79.430)
TyG-BMI				
Q1		Ref		Ref
Q2	< 0.001	5.766 (2.551, 13.031)	< 0.001	6.031 (2.658, 13.685)
Q3	< 0.001	19.682 (9.073, 42.695)	< 0.001	21.093 (9.627, 46.217)
Q4	< 0.001	44.353 (20.618, 95.413)	< 0.001	45.527 (20.842, 99.447)

Model 1: unadjusted; Model 2: adjusted for age, sex, history of hypertension.

**Figure 1:**
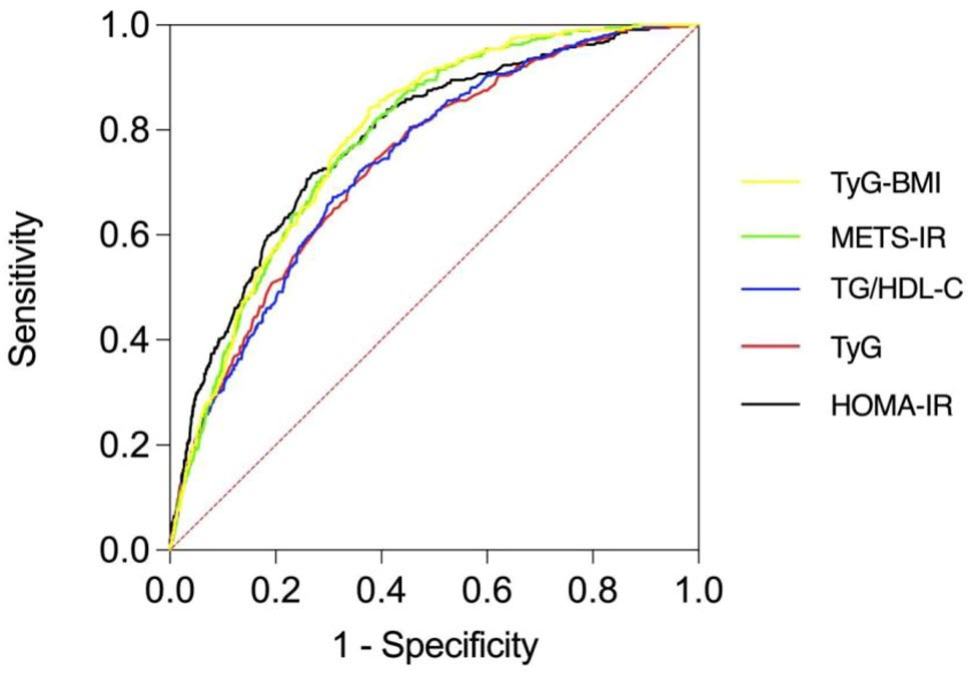
AUCs of 5 IR markers associated with NAFLD.

In the total population, the AUC of each IR marker was greater than 0.5 (P < 0.05), indicating specific predictive values for NAFLD. The AUC values decreased in the following order: TyG-BMI (0.788, 95% CI 0.766–0.811), METS-IR (0.783, 95% CI 0.760–0.806), HOMA-IR (0.782, 95% CI 0.756–0.808), TyG (0.738, 95% CI 0.711–0.765), TG/HDL-c (0.738, 95% CI 0.711–0.764). The P value greater than 0.05 for the difference in AUC between any two of the first three IR markers indicates lack of statistically significant differences between TyG-BMI, METS-IR and HOMA-IR. The AUCs of TyG-BMI, METS-IR and HOMA-IR were significantly higher than those of TyG and TG/HDL-c (P < 0.05), respectively. Thus, the ability of TyG-BMI, METS-IR and HOMA-IR to detect NAFLD was significantly higher than that of the other 2 IR makers in the overall non-diabetic population. The AUC of TyG-BMI was the largest (Table 3, Fig. 1).

**Table 3:** AUCs of 5 IR Markers in relation to NAFLD.

Variables	AUC (95% CI)	Sensitivity (%)	Specificity (%)	Youden
HOMA-IR	0.782 (0.756–0.808)*^&^	71.8	72.9	0.447
TyG-BMI	0.788 (0.766–0.811)*^&^	84.2	62.3	0.465
METS-IR	0.783 (0.760–0.806)*^&^	82.2	60.9	0.431
TyG	0.738 (0.711–0.765)	74.6	61.2	0.358
TG/HDL-c	0.738 (0.711–0.764)	67.2	69.2	0.364

*P < 0.05 for AUC difference from TyG.

^&^P < 0.05 for AUC difference from TG/HDL-c.

### The association of 5 IR markers with NAFLD in non-obese populations

The relationship between the 5 IR markers and NAFLD in the non-obese population was similar to that in the overall population with the risk of NAFLD increasing with the TyG, TyG-BMI, HOMA-IR, TG/HDL-c and METS-IR quartiles. Based on ORs (OR 58.288, 95% CI 13.729–247.468, and OR 33.071, 95% CI 10.110–108.177), the top two IR markers were METS-IR and TyG-BMI, which were most pronounced after adjustment for age, sex, and history of hypertension in Model 2 (Table 4).

**Table 4:** Logistic regression analysis of 5 IR markers and NAFLD in non-obese population.

	Model 1		Model 2	
	P value	OR (95% CI)	P value	OR (95% CI)
HOMA-IR				
Q1		Ref		Ref
Q2	0.497	1.356 (0.563, 3.266)	0.497	1.356 (0.563, 3.269)
Q3	0.017	2.628 (1.190, 5.806)	0.018	2.601 (1.175, 5.759)
Q4	< 0.001	8.060 (3.918, 16.583)	< 0.001	7.865 (3.793, 16.308)
TG/HDL-c				
Q1		Ref		Ref
Q2	0.025	3.619 (1.178, 11.124)	0.026	3.603 (1.169, 11.103)
Q3	0.016	3.959 (1.299, 12.070)	0.016	3.971 (1.296, 12.169)
Q4	< 0.001	22.241 (8.002, 61.821)	< 0.001	22.774 (8.108, 63.967)
TyG				
Q1		Ref		Ref
Q2	0.579	1.306 (0.508, 3.355)	0.583	1.303 (0.506, 3.352)
Q3	0.032	2.507 (1.081, 5.816)	0.039	2.434 (1.046, 5.663)
Q4	< 0.001	10.511 (4.946, 22.336)	< 0.001	10.188 (4.767, 21.776)
METS-IR				
Q1		Ref		Ref
Q2	0.025	5.687 (1.250,25.877)	0.017	6.402 (1.400,29.275)
Q3	0.001	12.936 (3.030,55.235)	< 0.001	16.466 (3.807,71.217)
Q4	< 0.001	41.513 (10.062,171.271)	< 0.001	58.288 (13.729,247.468)
TyG-BMI				
Q1		Ref		Ref
Q2	0.481	1.678 (0.397, 7.083)	0.445	1.755 (0.414, 7.445)
Q3	< 0.001	8.598 (2.561, 28.867)	< 0.001	9.545 (2.811, 32.405)
Q4	< 0.001	29.986 (9.324, 96.430)	< 0.001	33.071 (10.110, 108.177)

Model 1: unadjusted; Model 2: adjusted for age, sex, and history of hypertension.

**Figure 2:**
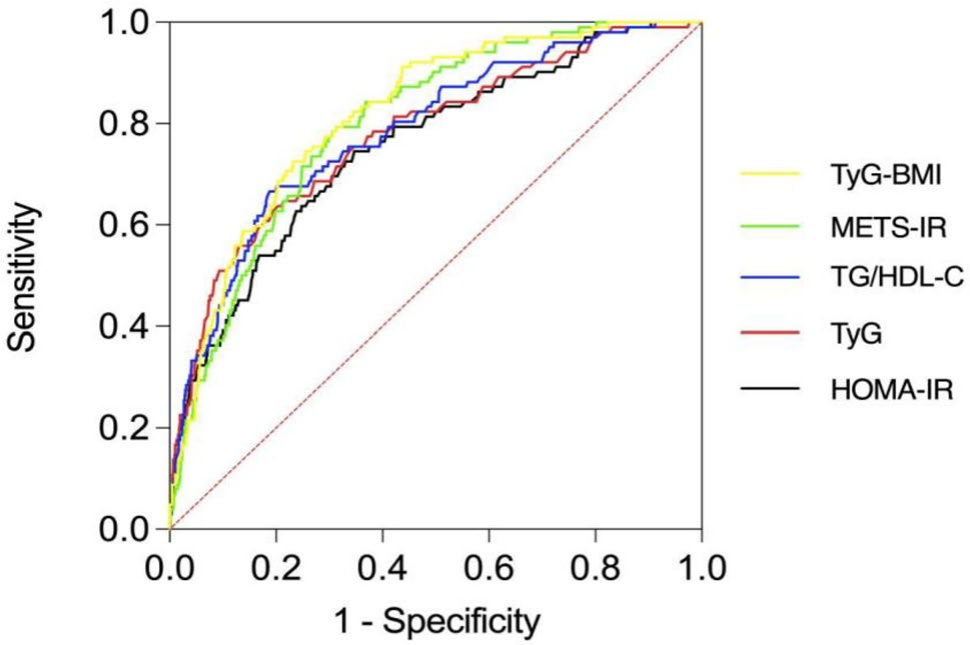
AUCs of 5 IR markers in relation to non-obese NAFLD.

The AUC of TyG-BMI exceeded 0.80 and was the largest. The Youden index was 0.492, indicating good predictive performance and diagnostic accuracy. The AUCs of the 5 IR markers were TyG-BMI (0.817, 95% CI 0.778–0.856), METS-IR (0.800, 95% CI 0.760–0.839), TyG (0.778, 95% CI 0.728–0.828), TG/HDL-c (0.784, 95% CI 0.737–0.832), and HOMA-IR (0.755, 95% CI 0.704–0.805). The AUC of TyG-BMI was larger than that of TyG, TG/HDL-c, and HOMA-IR (P < 0.05). The difference between any two AUC values involving METS-IR, TyG, TG/HDL-c and HOMA-IR was not statistically significant (P > 0.05). This means that TyG-BMI is better than the other four IR markers in detecting NAFLD in non-obese people (Table 5, Fig. 2).

**Table 5:** AUCs of 5 IR markers in relation to non-obese NAFLD.

Variables	AUC (95% CI)	Sensitivity (%)	Specificity (%)	Youden
TyG-BMI	0.817 (0.778–0.856)	72.5	76.7	0.492
METS-IR	0.800 (0.760–0.839)	79.4	68.8	0.482
TyG	0.778 (0.728–0.828)*	63.7	80.5	0.442
TG/HDL-c	0.784 (0.737–0.832)*	66.7	81.7	0.484
HOMA-IR	0.755 (0.704–0.805)*	74.5	65.4	0.399

*P < 0.05 for AUC difference from TyG-BMI.

### The association of 5 IR markers with NAFLD in obese populations

The risk of NAFLD was increased with the increase of HOMA-IR, TyG, TG/HDL-c, TyG-BMI and METS-IR quartiles. The highest ORs were found in the highest quartile group of HOMA-IR in Models 1 and 2 (Table 6).

**Table 6:** Logistic regression analysis of 5 IR Markers and NAFLD in obese population.

	Model 1		Model 2	
	P value	OR (95% CI)	P value	OR (95% CI)
HOMA-IR				
Q1		Ref		Ref
Q2	0.001	2.739 (1.530, 4.902)	0.001	2.753 (1.534, 4.939)
Q3	< 0.001	6.179 (3.556, 10.737)	< 0.001	6.199 (3.553, 10.816)
Q4	< 0.001	11.090 (6.423, 19.147)	< 0.001	10.711 (6.170, 18.593)
TG/HDL-c				
Q1		Ref		Ref
Q2	< 0.001	2.421 (1.484, 3.950)	0.001	2.376 (1.450, 3.896)
Q3	< 0.001	3.477 (2.161, 5.595)	< 0.001	3.560 (2.200, 5.763)
Q4	< 0.001	4.474 (2.795, 7.161)	< 0.001	4.713 (2.917, 7.614)
TyG				
Q1		Ref		Ref
Q2	0.002	2.215 (1.355, 3.621)	0.001	2.261 (1.377, 3.712)
Q3	< 0.001	3.801 (2.369, 6.100)	< 0.001	3.799 (2.356, 6.126)
Q4	< 0.001	4.103 (2.559, 6.581)	< 0.001	4.225 (2.616, 6.822)
METS-IR				
Q1		Ref		Ref
Q2	0.005	2.086 (1.254, 3.470)	0.005	2.080 (1.247, 3.468)
Q3	< 0.001	3.895 (2.400, 6.322)	< 0.001	3.874 (2.375, 6.319)
Q4	< 0.001	5.399 (3.344, 8.717)	< 0.001	5.117 (3.141, 8.336)
TyG-BMI				
Q1		Ref		Ref
Q2	0.046	1.665 (1.009, 2.749)	0.040	1.695 (1.024, 2.805)
Q3	< 0.001	3.454 (2.159, 5.525)	< 0.001	3.341 (2.083, 5.358)
Q4	< 0.001	4.874 (3.065, 7.752)	< 0.001	4.582 (2.866, 7.325)

Model 1: unadjusted; Model 2: adjusted for age, sex, and history of hypertension.

**Figure 3:**
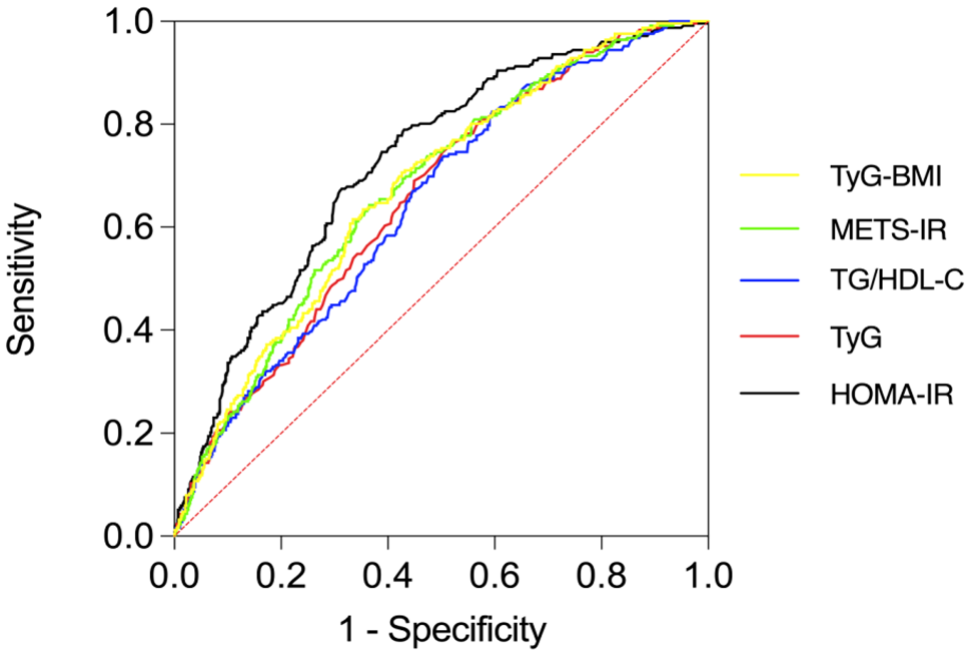
AUCs for 5 IR markers associated with obese NAFLD.

In the obese population, the AUC of HOMA-IR was the largest (0.724, 95% CI 0.689–0.760) and differed significantly from that of the other 4 IR markers (P < 0.05). The AUCs of TyG-BMI, METS-IR,TyG and TG/HDL-C were 0.675 (95% CI 0.638–0.712), 0.674 (95% CI 0.637–0.712), 0.656 (95% CI 0.618–0.693) and 0.645 (95% CI 0.607–0.684), respectively. The difference in AUC values between any two of them had no statistical significance (P > 0.05). This suggests that HOMA-IR has a better predictive value for NAFLD in obese individuals (Table 7, Fig. 3).

**Table 7:** AUCs for 5 IR markers associated with obese NAFLD.

Variables	AUC (95% CI)	Sensitivity (%)	Specificity (%)	Youden index
HOMA-IR	0.724 (0.689–0.760)	67.5	68.9	0.364
TyG-BMI	0.675 (0.638–0.712)*	63.5	64.6	0.281
METS-IR	0.674 (0.637–0.712)*	64.3	63.5	0.278
TyG	0.656 (0.618–0.693)*	74.6	50.1	0.247
TG/HDL-c	0.645 (0.607–0.684)*	73.8	49.9	0.237

*P < 0.05 for AUC difference from HOMA-IR.

## Discussion

The study findings suggest that IR is strongly associated with the risk of NAFLD in patients with obese and non-obese NAFLD. All IR markers were significantly higher in the NAFLD group than in the non-NAFLD group in the overall study population. After adjusting for confounding factors using logistic regression, the ORs for TyG, TyG-BMI, TG/HDL-c, METS-IR, and HOMA-IR tended to increase with increasing quartile levels, in the total population, as well as in obese and non-obese subgroups. In the non-obese subgroup, the AUC (0.817, 95% CI 0.778–0.856) of TyG-BMI was the highest among all 5 IR markers, suggesting that TyG-BMI has a better predictive value for NAFLD in non-diabetic and non-obese subjects. In the obese subgroup, the AUC of HOMA-IR was the largest (AUC 0.724, 95% CI 0.689–0.760) compared with the other four IR markers (P < 0.05). Thus, HOMA-IR is a better predictor for NAFLD in non-diabetic obese subjects compared with other IR markers.

Our study corroborates previous reports of IR markers associated with the risk of NAFLD^15,19,20^. However, in various studies, the association between IR markers and the risk of NAFLD has shown inconsistent results. Le et al. found that TyG-BMI and METS-IR demonstrated superior discriminatory ability for NAFLD compared to TyG and TG/HDL-c in subjects aged ≥ 60 years^15^. However, the study did not differentiate between obese and non-obese participants. A study found that elevated METS-IR was independently associated with new-onset NAFLD in non-obese populations^21^. Fedchuk et al. found that TyG had a significant diagnostic accuracy for the presence of steatosis with an AUC of 0.90^22^. Zhang et al. analysed 6809 Chinese subjects with BMI < 25.0 kg/m^2^ and reported that TyG-BMI was more accurate in identify NAFLD than TyG, with an AUC of 0.835^23^, However, HOMA-IR and TG/HDL-c were not compared in that study. In a study of 826 individuals with type 2 diabetes, TyG-BMI (AUC 0.727) was found to be a better predictor of NAFLD than TyG, HOMA-IR, or TG/HDL-c, the predictive value of TyG-BMI for NAFLD in obese subjects was higher than in non-obese subjects^19^. Although the results of these studies differed, they consistently showed a strong correlation between IR markers and NAFLD. These differences may be attributed to the different populations, races, and diagnostic methods used. As commonly used diagnostic tools, a finding suggests variation in the prevalence of NAFLD diagnosed by CT and US^24^.

In our study, the association between IR markers and obese and non-obese NAFLD does not show a consistent pattern. The role of TyG-BMI in predicting non-obese NAFLD was better than the other 4 IR markers. HOMA-IR is more effective in predicting obese NAFLD. The reason for this distinction is not clear. TyG-BMI is calculated using FPG, TG and BMI. The diagnostic value of TyG-BMI in non-obese individuals may be superior as it is based on body fat distribution. Studies suggest that normal BMI in non-obese people still indicates an independent risk factor for NAFLD^23,25^, which may be due to the higher body fat content of individuals with non-obese NAFLD than in non-obese non-NAFLD subjects^25^.

The study has several advantages. First, NAFLD was investigated instead of metabolic-associated fatty liver disease (MAFLD) in this study. The diagnosis of NAFLD and MAFLD is based on pathological hepatic steatosis and radiographic diffuse fatty liver. However, the diagnostic criteria for MAFLD are also based on overweight and obesity, T2DM, or the presence of two or more metabolic risk abnormalities^26^. This may lead to the exclusion of certain lean or non-obese individuals^27^. Second, this study compared the predictive value of insulin-based IR marker (HOMA-IR) and non-insulin-based IR markers (TyG, TyG-BMI, TG/HDL-c, and METS-IR) for NAFLD in a large non-diabetic population and in obese and non-obese sub-groups. To the best of our knowledge, this study represents the first comparative analysis of five IR markers like this. Third, the CT results of this study were diagnosed by two experienced doctors, thus minimising the diagnostic deviation.

However, the study also has limitations. First, the study was conducted in a single medical centre. Second, CT was used in this study to diagnose NAFLD, which may miss diagnosis compared with liver biopsy.

## Conclusion

The risk of NAFLD is positively associated with elevations in both insulin-based and non-insulin-based IR markers, including HOMA-IR, TyG, TyG-BMI, TG/HDL-c, and METS-IR in non-diabetic population. Results of sub-group analyses indicate that TyG-BMI (AUC 0.817, 95% CI 0.778–0.856) and HOMA-IR (AUC 0.724, 95% CI 0.689–0.760) are more effective than the other four IR markers for detection of non-obese and obese NAFLD, respectively.

## Data Availability

The data that support the findings of this study are available on request from the corresponding author. The data are not publicly available due to privacy or ethical restrictions.

## References

[1] Powell EE, Wong VW, Rinella M (2021). Non-alcoholic fatty liver disease. The Lancet.

[2] Zhou J (2020). Epidemiological features of NAFLD from 1999 to 2018 in China. Hepatology.

[3] Younossi Z (2018). Global burden of NAFLD and NASH: Trends, predictions, risk factors and prevention. Nat. Rev. Gastroenterol. Hepatol..

[4] Li J (2019). Prevalence, incidence, and outcome of non-alcoholic fatty liver disease in Asia, 1999–2019: A systematic review and meta-analysis. Lancet Gastroenterol. Hepatol..

[5] Zou B, Yeo YH, Nguyen VH, Cheung R, Ingelsson E, Nguyen MH (2020). Prevalence, characteristics and mortality outcomes of obese, nonobese and lean NAFLD in the United States, 1999–2016. J. Intern. Med..

[6] Hagström H (2018). Risk for development of severe liver disease in lean patients with nonalcoholic fatty liver disease: A long-term follow-up study. Hepatol. Commun..

[7] Kim D, Kim WR (2017). Nonobese fatty liver disease. Clin. Gastroenterol. Hepatol..

[8] Dorairaj V, Sulaiman SA, Abu N, Abdul Murad NA (2022). Nonalcoholic fatty liver disease (NAFLD): Pathogenesis and noninvasive diagnosis. Biomedicines.

[9] Watt MJ, Miotto PM, Nardo WD, Montgomery MK (2019). The liver as an endocrine organ-linking NAFLD and insulin resistance. Endocr. Rev..

[10] Khan RS, Bril F, Cusi K, Newsome PN (2019). Modulation of insulin resistance in nonalcoholic fatty liver disease. Hepatology.

[11] Koo DJ (2021). Baseline homeostasis model assessment of insulin resistance associated with fibrosis progression in patients with nonalcoholic fatty liver disease without diabetes: A cohort study. PLoS ONE.

[12] DeFronzo RA, Tobin JD, Andres R (1979). Glucose clamp technique: A method for quantifying insulin secretion and resistance. Am. J. Physiol..

[13] Matthews DR, Hosker JP, Rudenski AS, Naylor BA, Treacher DF, Turner RC (1985). Homeostasis model assessment: Insulin resistance and β-cell function from fasting plasma glucose and insulin concentrations in man. Diabetologia.

[14] Silva CC, Zambon MP, Vasques ACJ, Camilo DF, Antonio MRGM, Geloneze B (2023). The threshold value for identifying insulin resistance (HOMA-IR) in an admixed adolescent population: A hyperglycemic clamp validated study. Arch. Endocrinol. Metab..

[15] Li H (2023). Relationship between six insulin resistance surrogates and nonalcoholic fatty liver disease among older adults: A cross-sectional study. Diabetes Metab. Syndr. Obes..

[16] Hu PF (2021). The presence of NAFLD in nonobese subjects increased the risk of metabolic abnormalities than obese subjects without NAFLD: A population-based cross-sectional study. Hepatobiliary Surg. Nutr..

[17] Zhang Z, Zhang L, Jiang W, Du T, Yuan G (2022). Non-obese NAFLD had no better cardio-metabolic risk profile than obese NAFLD in type 2 diabetic patients. Cardiovasc. Diabetol..

[18] Qian T, Sheng X, Shen P, Fang Y, Deng Y, Zou G (2023). Mets-IR as a predictor of cardiovascular events in the middle-aged and elderly population and mediator role of blood lipids. Front. Endocrinol..

[19] Li N (2022). Value of the triglyceride glucose index combined with body mass index in identifying non-alcoholic fatty liver disease in patients with type 2 diabetes. BMC Endocr. Disord..

[20] Peng H (2023). Prediction of MAFLD and NAFLD using different screening indexes: A cross-sectional study in US adults. Front. Endocrinol..

[21] Cai X (2022). Dose-response associations of metabolic score for insulin resistance index with nonalcoholic fatty liver disease among a nonobese Chinese population: Retrospective evidence from a population-based cohort study. Dis. Mark..

[22] Fedchuk L (2014). Performance and limitations of steatosis biomarkers in patients with nonalcoholic fatty liver disease. Aliment Pharmacol. Ther..

[23] Zhang S (2017). Triglyceride glucose-body mass index is effective in identifying nonalcoholic fatty liver disease in nonobese subjects. Medicine.

[24] Ito Y (2023). Prevalence of non-alcoholic fatty liver disease detected by computed tomography in the general population compared with ultrasonography. Intern. Med..

[25] Das K (2010). Nonobese population in a developing country has a high prevalence of nonalcoholic fatty liver and significant liver disease. Hepatology.

[26] Eslam M (2020). The Asian Pacific Association for the study of the liver clinical practice guidelines for the diagnosis and management of metabolic associated fatty liver disease. Hepatol. Int..

[27] Ng CH, Huang DQ, Nguyen MH (2022). Nonalcoholic fatty liver disease versus metabolic-associated fatty liver disease: Prevalence, outcomes and implications of a change in name. Clin. Mol. Hepatol..

